# Forethought in Youth with Attention Deficit/Hyperactivity Disorder: An fMRI Study of Sex-Specific Differences

**DOI:** 10.1155/2016/6810215

**Published:** 2016-07-27

**Authors:** H. Poissant, L. Rapin, S. Chenail, A. Mendrek

**Affiliations:** ^1^Department of Education and Pedagogy, UQAM, Montreal, QC, Canada H2L 2C4; ^2^Department of Linguistics, UQAM, Montreal, QC, Canada H2L 2C4; ^3^Department of Psychology, Bishop's University, Sherbrooke, QC, Canada J1M 1Z7

## Abstract

*Objective*. The majority of studies investigating neurocognitive processing in attention deficit/hyperactivity disorder (ADHD) have been conducted on male participants. Few studies evaluated females or examined sex differences. Among various cognitive anomalies in ADHD, deficit in forethought seems particularly important as children with ADHD often fail to adequately use previous information in order to prepare for responses. The main goal of this study was to assess sex-specific differences in behavioral and neural correlates of forethought in youth with ADHD.* Methods*. 21 typically developing (TD) youth and 23 youth with ADHD were asked to judge whether two pictures told a congruent or incongruent story. Reaction time, performance accuracy, and cerebral activations were recorded during functional magnetic resonance imaging (fMRI).* Results*. Significant sex-specific differences in cerebral activations appeared, despite equivalent performance. Relative to the boys TD participants, boys with ADHD had extensive bilateral frontal and parietal hypoactivations, while girls with ADHD demonstrated more scattered hypoactivations in the right cerebral regions.* Conclusion*. Present results revealed that youth with ADHD exhibit reduced cerebral activations during forethought. Nevertheless, the pattern of deficits differed between boys and girls, suggesting the use of a different neurocognitive strategy. This emphasizes the importance of including both genders in the investigations of ADHD.

## 1. Introduction

Attention deficit/hyperactive disorder (ADHD) is a neurodevelopmental disorder defined by persistent inattention and/or hyperactivity/impulsivity [1]. The disorder negatively impacts social, academic, or occupational functioning. Most of the studies have been conducted on male participants resulting in a limited knowledge regarding sex and gender differences in ADHD [2]. The higher prevalence of ADHD in boys could partly explain this gap in the literature [3], but we need more information regarding ADHD females, especially in the realm of neurocognitive dysfunction. Thus, the present study was designed to examine neural sex differences in ADHD.

The expression of ADHD appears to be sexually dimorphic. Boys seem to exhibit more hyperactivity, inattention, impulsivity, and externalizing problems. Girls generally show more internalizing problems (depression, anxiety) and intellectual impairment [4, 5]. Larger deficits in inhibition have also been reported in males [6]. Differences in age of onset and differences in symptoms could be due to neurochemical and hormonal factors. For example, boys show a significant increase in dopamine (DA) receptor density before and during puberty, which could contribute to more hyperactivity symptoms at an earlier stage [7]. In comparison, girls demonstrate a later increase in DA receptors related to an increase in estrogen during puberty [7].

Several executive dysfunctions have been found in children with ADHD, including difficulties with prioritizing, time management, and initiating and completing tasks as well as difficulty in shifting cognitive sets and poor working memory (WM) [8]. Working memory, defined as the ability to retain information for prospective execution of an action [9], seems specifically affected in ADHD [3]. A meta-analysis indicated that children with ADHD scored lower than control children on verbal storage, verbal central executive, visual-spatial storage, and visual-spatial central executive [10]. Another meta-analysis [11] found significant patterns of hypoactivity in ADHD, which affected the anterior cingulate cortex (ACC), dorsolateral and inferior prefrontal cortex (PFC), basal ganglia, thalamus, and portions of the parietal cortex. Other findings also point to deficits in the neural activity of the frontostriatal and frontoparietal pathways [12]. Studies on inhibition alone indicate hypoactivation, especially pronounced in the PFC, and greater activity in the medial frontal gyrus and the parietal paracentral lobule in individuals with ADHD [11].

Only a few neuroimaging studies on ADHD have included females. The percentage of females across all studies has been estimated to be less than 20% [2] while the male to female ratio among ADHD is estimated to be 2.28 : 1 [13]. Valera and colleagues (2010) [14] found that while performing a verbal WM task, males with ADHD showed significantly less activation in the right frontal, right temporal, left occipital, cerebellar, and some subcortical regions. However, no differences in activation were found between females with and without ADHD. Similar results were obtained in a subsequent fMRI study [15], while the electroencephalography (EEG) investigation revealed markedly different EEG profiles in boys and girls with ADHD [16].

*Forethought* is a prospective function of WM related to the ability to conjecture possible future related events [17]. Children with ADHD often fail to adequately use previous information in order to prepare for upcoming responses. One study found that children with ADHD showed diminished activation in the ventral PFC and ACC when having to react to a stimulus that was not expected from a past stimulus [18]. More recently, Poissant et al. (2012) [19] also found hypoactivation of the PFC in youth with ADHD and higher reaction times during a task requiring forethought.

In the present study we examined neural sex differences in ADHD during a forethought task. Based on previous findings, it was expected that boys with ADHD would demonstrate poorer performance and greater cerebral hypoactivation during the task relative to girls with ADHD.

## 2. Method

### 2.1. Participants

Forty-four children (21 control (12 girls) and 23 with ADHD (7 girls); age range: 7–15 years old) composed the two groups of this cross-sectional study. Control participants (TD) were recruited through schools in the Montreal area. Participants with ADHD were recruited through a specialized clinic for ADHD in Montreal. All participants with ADHD had received a diagnosis by a specialized medical team based on DSM-IV-TR criteria (APA, 2000). In the following weeks, all parents filled out the Risk Factor Questionnaire (RFQ) [20] to confirm diagnosis in participants with ADHD as well as to exclude signs of the disorder in TD. Recruitment of patients was done over two and a half years, from 2009 to 2012. In addition, both sustained and selective attention were measured with the Continuous Performance Task (CPT) [21]. The ability to shift sets was measured by the Wisconsin Card Sorting Test (WCST) [22] and working memory with the Auditory Consonant Trigrams (ACT) test for children [23]. Mean composite IQ scores (K-BIT) [24] were also assessed in both groups. All adolescents were assessed in two sessions: neuropsychological and cerebral imagery while they were deprived of methylphenidate (MPH) for at least 24 hr. Children with motor, auditory or vision deficits, central nervous system abnormalities, and major health problems were excluded.

### 2.2. Functional Magnetic Resonance Imaging (fMRI)

The neural activation was assessed with fMRI. While in the scanner, participants were asked to judge whether two consecutive pictures told a congruent or incongruent story. There were 56 stories: 28 congruent (e.g., a picture of a girl grabbing a pint of milk followed by a picture of the same girl pouring milk in cereal: COherent condition) and 28 noncongruent (e.g., a picture of a girl grabbing a pint of milk followed by a picture of the same girl pouring water into a flower vase: INCOherent condition). The number of correct responses for each condition and the reaction time for each item were recorded. An fMRI blood oxygenation level dependent (BOLD) signal was recorded during performance of the task. A single-shot, gradient-recalled echo-planar imaging sequence (repetition time = 2000 ms, echo time = 30 ms, flip angle = 90 degrees, and matrix 64 × 64 voxels) was used on a MRI Siemens TRIO system at 3.0 Tesla at Institut Universitaire Gériatrique de Montréal (IUGM). The images consisted of 32 continuous axial slices, with a 3 mm × 3 mm in-plane resolution. The slice thickness was 3 mm. During the run, 270 volumes were continuously acquired over a total duration of 540 seconds. A high-resolution T1-weighted scan (1 mm^3^ voxel size) was provided for each subject for anatomical coregistration. Prospective acquisition correction was applied to the images for better correction of head movement.

### 2.3. Data Analysis

Analyses of standardized neurological tests were done on the *T*-scores of all CPT and WCST measures and on raw scores for ACT. A 2*∗*2 ANOVA with the diagnosis and sex as between-subjects factors was performed on each measure. Interaction effects and pairwise comparisons were explored using the Bonferroni correction with the alpha level set to 0.05. A log10 transformation was necessary to normalize the forethought data distribution and a 2*∗*2 ANCOVA with age as a covariate was performed for the number of correct responses (Hits), number of missed responses (Misses), reaction time (RT), and reaction time per correct responses (RT/Hit) for both the CO and INCO conditions. Previous to performing ANOVAs and ANCOVAs, assumptions of normality, homogeneity of variance, and presence of outliers were checked for each test and none were violated.

The fMRI data were processed using statistical parametric mapping (SPM8, Wellcome Department of Cognitive Neurology, London, UK). Individual cerebral activation of INCO > CO contrast served as the measure of forethought. A 2 × 2 ANCOVA was conducted in SPM8, with diagnosis and sex as independent factors and age as the covariate, to assess level of neural activation related to forethought. Because the literature is quite dispersed regarding the neural deficits in ADHD (except for the frontostriatal network hypothesis), a whole brain analysis was conducted. The effects of diagnosis and sex were investigated in the contrasts [TD > ADHD] and [ADHD > TD],and interaction effects in the following simple contrasts: [TD boys > ADHD boys], [TD girls > ADHD girls], [ADHD boys > TD boys], and [ADHD girls > TD girls]. Statistical tests were performed for group comparisons at a threshold level of *p* = 0.001 uncorrected for multiple comparisons with a cluster threshold of 10 voxels. This more liberal threshold was used because the expected effects are broad but weak [25].

## 3. Results

### 3.1. Neuropsychological Profiles

Mean composite IQ scores (K-BIT) [24] varied between 103 and 114 and no IQ differences were found between diagnostic groups, sex or group-by-sex interaction (Table 1). Most neuropsychological measures indicated that boys with ADHD were in the mildly atypical clinical range pointing mostly to inattentive symptoms (CPT: omission, Hit RT, Hit RT SE). Their larger *T*-scores (> 60) on WCST also suggested lower mental flexibility (Table 2). Analysis of CPT indicated several main effects and sex-by-diagnostic interactions. A significant difference of omission was found between boys with ADHD and TD boys (*p* = 0.017) but not between TD girls and girls with ADHD. Regarding Hit RT, higher scores for boys with ADHD compared to girls with ADHD were observed (*p* = 0.01). For the Hit RT SE, the ADHD groups obtained higher scores and, specifically, boys with ADHD obtained significantly higher scores compared to TD boys (*p* = 0.016) and to TD girls (*p* = 0.037). The analysis of Hit RT Block change revealed that boys with ADHD obtained a higher score compared to girls with ADHD (*p* = 0.024) but no difference appeared between girls and boys in the TD group. The same pattern of results occurred with Standard Errors for reaction time over Blocks (Hit SE Block change). Boys with ADHD displayed larger scores compared to girls with ADHD (*p* = 0.004) and to TD girls (*p* = 0.003). A main effect of diagnostic was found for the Standard Error of reaction time slope over different interstimulus intervals (HIT SE ISI change); youth with ADHD demonstrated less consistency compared to TD groups (*p* = 0.006), a tendency more acute in boys with ADHD. The ACT analysis revealed a lesser performance for the groups with ADHD (*p* = 0.013) but no sex difference nor interaction between sex^*∗*^diagnostic group was observed. No subtest of WCST was statistically significant in terms of group differences (for brevity, we did not include these data in Table 2).

### 3.2. Forethought Performance

Behavioral performance results are presented in Table 3. No significant difference was found in the number of correct answers. However, reaction times (RT) analyses revealed group differences. For the CO condition, the TD group provided systematically faster responses (RT) (*p* = 0.003) and faster RT per hit (*p* = 0.008) compared to the group with ADHD. For the INCO condition, the TD group demonstrated marginally faster RT Inco/Hits compared to the ADHD group (*p* = 0.073). Although neither sex nor interaction effects were found, boys with ADHD showed systematically longer reaction times compared to girls with ADHD.

### 3.3. Neural Correlates of Forethought

Cerebral activation findings are presented in Table 4. The diagnostic group contrast (TD > ADHD) showed significantly more activation in the right parietal postcentral gyrus (BA 1), left cingulate gyrus (BA 24), right middle frontal gyrus (MFG, BA 6), and the right culmen (cerebellum). No significant activation was observed in the opposite contrast (ADHD > TD). Relative to TD boys, boys with ADHD had extensive bilateral frontal and parietal hypoactivation, including bilateral superior frontal gyrus (BA 6), left inferior parietal lobule (IPL; BA 40), and right superior parietal lobule (SPL; BA 7). Boys with ADHD were also found to activate significantly more the right amygdala and left superior temporal gyrus than TD boys. Girls with ADHD demonstrated more scattered neural hypoactivation in comparison to TD girls including the right culmen, the right postcentral gyrus (BA 2), right middle temporal gyrus (MTG; BA 21), left claustrum (basal ganglia), and right inferior frontal gyrus (BA 47-10). There was no significant activation for the opposite contrast (ADHD girls > TD girls).

## 4. Discussion

Our expectations that ADHD boys would demonstrate more deficits than ADHD girls in sustained and selective attention and in cerebral activation during forethought were partially supported by the present study. Indeed, ADHD boys expressed greater inattentiveness, greater impulsivity, and lesser vigilance than TD boys, while ADHD girls did not differ from TD girls. Boys with ADHD also showed greater variability. Overall, WM capacities were lower in ADHD relative to TD group, confirming previous reports [3, 10]. Moreover, diagnosis by sex interactions observed on several occasions were compatible with studies reporting that boys with ADHD usually show more severe inattention symptoms [4] and greater executive deficits than girls with ADHD [15].

### 4.1. Forethought

The ADHD participants showed a tendency to be slower, but as accurate as the TD group on the forethought task. The forethought slowness was apparent especially in boys and was coherent with the CPT findings. As expected, extensive brain activation was observed in the TD group, including parietal and prefrontal regions as well as part of the cingulate gyrus and the cerebellum, during the forethought task performance. In contrast, the ADHD group showed no neural activation during forethought. This finding is consistent with reports of attenuated cerebral activation in ADHD tapping cognitive control [26, 27], working memory [2], and inhibition and vigilance/attention [28]. Overall, our study confirms the hypothesis of impairment in the frontal-parietal pathway in ADHD [11, 12, 28]. Lateral portions of the PFC are part of the attentional network [29] and thus it is plausible that its malfunction is responsible for the encountered forethought slowness. Moreover, cerebral hypoactivation in ADHD youth is in accordance with their greater inattention and lack of vigilance, as well as weaker working memory.

### 4.2. Boys with ADHD

TD boys showed more extensive bilateral frontal activation relative to boys with ADHD, including the premotor cortex and the supplementary motor area (SMA). Our results are compatible with findings observed in TD adolescent groups [11, 19, 28] although no sex differentiation was made in these studies. Premotor cortex and SMA are involved in motor sequencing and planning [30], working memory [31], and visuospatial attention [32]. All these functions are essential for optimal performance of forethought. Moreover, hypoactivation of the SMA and the superior frontal gyrus has been accounted for motoric hyperactivity in ADHD [28].

TD boys also exhibited greater activation in the bilateral parietal cortex, left IPL and right SPL, relative to ADHD boys. The SPL is involved in visual representation of movements [33] and in WM related to motor sequence performance [34]. Thus, hypoactivation of SPL in boys with ADHD could have contributed to their impairment of forethought due to its link with WM. Similar results were observed by Dickstein et al. (2006) [11] where controls demonstrated significantly greater activation relative to ADHD in the left prefrontal and bilateral parietal lobes (IPL and SPL). The inferior part of SPL is related to inhibition, WM, and attention processes [28], all components of forethought. This region is part of the ventral attention network (VAN) that supports reorientation to behaviorally relevant external stimuli [35]. Thus, a deficient engagement of the VAN in ADHD may account for difficulties in adequately shifting attention to salient external stimuli [36]. Moreover, hypoactivation in VAN underpins ADHD-related deficits in detecting irregularities in the environment [28]. These considerations are relevant considering the present impairment in forethought, which involves a reflection on an incongruent succession of events.

Furthermore, boys with ADHD were found to hyperactivate a region adjacent to the medial temporal lobe, which is a part of the default mode network (DMN). This region was previously found to be hyperactivated in children with ADHD [28]. Fluctuations in activation of the DMN tend to correlate negatively with fluctuations in networks activated during task performance, typically the frontoparietal attention network [37]. The current findings in ADHD boys corroborate this pattern of activation. Indeed, their lesser functioning of the frontoparietal network might have impacted the DMN, resulting in disruption of the ongoing forethought performance. Together with the CPT results, this finding supports the recent perspective suggesting a link between variability and intrusion of “task-negative brain network” [38]. According to this perspective, RT variability is related to increased activity in DMN. The present study also supports the increasing recognition of motivational and emotional dysfunction in models of ADHD [39]. Boys with ADHD possibly perceived the task as more difficult or unpleasant, as it required a certain amount of time staying still and attentive, so that they had to rely on more activation of the amygdala to provide sufficient motivation.

### 4.3. Girls with ADHD

TD girls demonstrated widespread cerebral activation and there were no differences found for the ADHD girls > TD girls contrast. TD girls activated large portions of the cerebellum (culmen), a region activated in response to temporally unexpected stimuli [18]. There is mounting evidence that the basal ganglia and the cerebellum might project directly to one another [40, 41]. Interestingly, both regions were activated in TD girls, but not in ADHD girls. Moreover, hypoactivation of the frontocerebellar network in girls with ADHD might have negatively impacted their ability to predict “when” events are going to occur and affect detection of violations of these predictions as suggested by previous studies [42, 43]. Because of its outputs to both the PFC and the basal ganglia, the cerebellum is in position to influence activity in circuits implicated in ADHD [42]. Moreover, because of its protracted development and sexual dimorphism, the cerebellum could play a crucial role in the neuropathology of girls with ADHD [44]. TD girls also activated the middle temporal gyrus (BA 21), a region activated in controls in the Durston et al. (2007) study [18], which investigated expectancy violation in ADHD, a condition similar to the incongruent situation of forethought.

TD girls also activated the claustrum, a region highlighted by Dickstein et al. (2006) [11] as being more activated in controls compared to participants with ADHD during EF performance. According to Crick and Koch (2005) [45], this part of basal ganglia plays a regulatory role in consciousness and cognition. Implication of the basal ganglia in conjunction with other regions might have helped TD girls in selecting the appropriate action and reject an incongruent action. Indeed the basal ganglia is believed to help in deciding among several possible behaviors at any given time [46, 47].

However, to fully establish the primacy of cerebral dysfunction in ADHD, future studies will need to employ a more comprehensive examination of executive function, using tasks known to produce consistent patterns of activity in other regions considered putative sources of dysfunction. Because the forethought task produced specific patterns of activity in boys and girls, our paradigm seems promising in further exploration of sex differences in the neural correlates of ADHD. Recent advances in brain imaging technology (e.g., diffusion tensor imaging and magnetoencephalography) allow us to examine more precisely and directly the relationships between brain regions during a cognitive task. A closer examination of the brain functional and anatomical connectivity would help in identifying entire neural networks underlying cognitive deficits in ADHD. Future research, including girls in the field of ADHD, promises exciting results linking the genomic, structural, and functional changes in the brain of individuals with ADHD. Advances in understanding of the ADHD neurobiology will hopefully help in developing new psychotherapeutic interventions and in identifying more targeted pharmacotherapies so that child psychiatrists can better manage their patients.

In conclusion, the present findings point to greater EF deficits in boys compared to girls with ADHD. Moreover, boys with ADHD exhibited cerebral hypoactivation in the frontoparietal network. On the other hand, they hyperactivated cerebral regions linked to motivation. Taken together, these findings indicate that boys with ADHD show decreased attention processing that seems compensated by the use of supplemental motivation to perform as accurately as their TD counterparts. However, this compensation comes with a time cost. Girls with ADHD displayed more dispersed decreased neural activation in the frontocerebellar and frontoparietal systems compared to the TD girls. This suggests that they rely on different circuits to make decisions about incongruent situations with a related time cost. Overall, the present study supports the hypothesis of different neuropsychological and neurofunctional profiles in boys and girls with ADHD. This brings to light the importance of considering both genders while investigating ADHD in order to better understand the disorder and propose clinical intervention more specific to the gender.

## Figures and Tables

**Table 1:** Age and IQ of participants with ADHD and typically developing youth (TD).

ADHD	Boys				Girls			
	Mean	SD	Min	Max	Mean	SD	Min	Max
Age	10	2.3	7	15	11	1.51	7	14
K-Bit_Voc	103	13.3	75	130	99	11.6	85	120
K-Bit_mat.	110	16.3	91	150	107	12.8	93	127
K-Bit_total	107	15.3	83	134	103	8	94	115

TD	Boys				Girls			
	Mean	SD	Min	Max	Mean	SD	Min	Max

Age	11	1.2	9	12	11	1.9	7	14
K-Bit_Voc	98	14.3	78	113	107	14.1	72	119
K-Bit_mat.	108	16.3	69	136	118	12.2	96	136
K-Bit_total	103	17.6	74	128	114	13.7	82	129

ANOVA	*F*: Diagnosis	*p*		*F*: Sex	*p*	*F*: Group × Sex		*p*

Age	0.70	0.408		0.72	0.402	0.96		0.332
K-Bit_Voc	0.21	0.651		0.31	0.541	2.40		0.129
K-Bit_mat.	0.69	0.410		0.54	0.465	1.69		0.200
K-Bit_total	0.54	0.466		0.61	0.439	2.60		0.114

*Note.* K-Bit_Voc, Kaufman brief intelligence test_Vocabulary; K-Bit_mat., Kaufman brief intelligence test_Matrices; TD, typically developing youth; ADHD, youth with attention deficit and hyperactivity disorders.

All *p*s are two-tailed.

**Table 2:** Neuropsychological profile of participants with ADHD and typically developing youth (TD).

	ADHD boys		ADHD girls		TD boys		TD girls		*F*-value		
ACT	Mean	SD	Mean	SD	Mean	SD	Mean	SD	*F*: group	*F*: sex	*F*: (G) × (S)
Total	32.13	11.2	44.50	8.4	48.44	8.3	48.50	15.9	5.35^*∗∗*^	2.00	1.86

CPT											
Omission	58.11	9.8	47.18	3.7	45.68	7.3	49.07	11.3	3.05	1.56	5.63^*∗*^
Commission	53.54	5.4	56.78	6.3	46.58	21	59.96	18.5	0.16	3.10	1.16
Hit RT	58.42	12.3	40.42	5.2	48.89	6.9	48.21	13.5	0.06	6.65^*∗*^	5.73^*∗*^
Hit RT SE	61.46	10.5	49.42	11.1	47.10	7.8	49.56	12.1	4.21^*∗*^	1.91	4.37^*∗*^
Hit RT ISI	62.83	16.1	51.24	9.5	53.18	12.6	52.05	12.7	0.97	2.01	1.36
Hit SE ISI	59.48	11.7	55.24	11.5	45.09	9.5	49.67	8.8	8.51^*∗∗*^	0.00	1.69
Hit RT Block	60.45	11.3	48.2	13.4	50.07	13.6	48.77	11.9	0.47	5.96^*∗*^	4.38^*∗*^
Hit SE Block	59.08	7.9	40.95	11.3	48.88	11.7	49.11	7.53	0.12	9.46^*∗*^	9.96^*∗*^

*Note.* ACT, Auditory Consonant Trigrams test; CPT, Continuous Performance Task; omission, number of times the subject fails to respond when target was presented; commission, number of response in absence of target; Hit RT, overall reaction time; Hit RT Block, slope of change in RTs over the six time blocks; Hit SE Block, slope of change in RTs standard errors over the six time blocks; Hit RT ISI, slope of change in RTs over the three interstimulus intervals; Hit SE ISI, slope of change in RTs SE over the three ISI; *F*: group corresponds to the Fisher test comparing youth with ADHD to youth without; *F*: sex corresponds to the Fisher test comparing boys to girls and *F*(G × S) corresponds to the Fisher measure observed for the interaction between the group and the sex.

^*∗*^ *p* < 0.05; ^*∗∗*^ *p* < 0.01; all *p*s are two-tailed.

**Table 3:** Forethought performance in participants with ADHD and typically developing (TD) youth.

Measures	ADHD boys		ADHD girls		TD boys		TD girls		2 × 2 ANCOVA		
	Mean	SD	Mean	SD	Mean	SD	Mean	SD	*F*: (G)	*F*: (S)	*F*: (G) × (S)
Co_hit	24.19	6.0	26.14	1.95	27.33	0.71	27	1.04	0.87	0.03	0.49
Co_miss	2.63	5.9	1.14	1.22	0.44	0.53	0.5	0.91	3.29	0.08	0.21
RT_Co	1412	256	1407	555	931.5	180	1150	270	10.02^*∗∗*^	2.49	0.97
RT Co/hit	74.94	69.8	55.15	26.7	34.07	6.48	42.73	10.3	7.93^*∗∗*^	0.87	1.39

Inco_hit	23.38	6.5	26	1.9	27.22	0.8	26.75	1.2	0.74	0.07	0.53
Inco_miss	2.81	6.5	1.14	1.5	0.33	0.5	0.92	1.1	0.98	0.12	0.57
RT_Inco	1474	605	1341	551	996.6	228	1255	310	2.93	1.86	1.36
RT Inco/hit	95.9	134	52.49	24.6	36.59	8.14	47.22	12.9	3.38^†^	0.40	1.88

*Note*. Co_hit, good answer in authorized delay for coherent condition; Inco_hit, good answer in authorized delay for incoherent condition; Co_miss, error in authorized delay for coherent condition; Inco_miss, error in authorized delay for incoherent condition; RT_Co, mean reaction time (ms.) for coherent condition; RT Co/hit, mean reaction time (ms.) per hit for coherent condition; RT_Inco, mean reaction time (ms) for incoherent condition; RT Inco/hit, mean reaction (ms.) per hit for incoherent condition; *F*: group corresponds to the Fisher test comparing youth with ADHD to youth without; *F*: sex corresponds to the Fisher test comparing boys to girls and *F*(G × S) corresponds to the Fisher measure observed for the interaction between the group and the sex.

^*∗∗*^ *p* < 0.01; † = 0.07; all *p*s are two-tailed.

**Table 4:** Brain activations during forethought: contrast INCO-CO in participants with ADHD and typically developing (TD) youth.

Comparison/region	*K* (voxel)	*Z* value	MNI			*p* value
			*x*	*y*	*z*	
TD-ADHD						
Right parietal postcentral gyrus, BA 1	124	4.42	66	−22	40	0.000
Left cingulate gyrus, BA 24	126	3.69	−3	−4	46	0.000
Right middle frontal gyrus, BA 6	92	4.10	27	−7	67	0.000
Right anterior lobe, culmen	64	3.62	30	−52	−32	0.000

ADHD-TD						
*No voxel activated*						

TD boys-ADHD boys						
Bilateral superior frontal gyrus, BA 6	3300	4.36	−18	−4	58	0.000
Left inferior parietal lobule, BA 40	158	3.81	−66	−34	31	0.000
Right superior parietal lobule, BA 7	110	3.22	12	−64	61	0.001

ADHD boys-TD boys						
Right amygdala	578	3.18	39	−4	−20	0.001
Left superior temporal gyrus	114	3.15	−39	8	−23	0.001

TD girls-ADHD girls						
Right cerebellum, culmen	549	3.61	69	−22	34	0.000
Right, postcentral gyrus, BA 2	180	3.25	30	−55	−29	0.001
Right middle temporal gyrus, BA 21	137	3.70	57	−55	−1	0.000
Left claustrum	38	3.25	−24	26	−8	0.001
Right frontal gyrus, BA 10	32	3.15	45	53	1	0.001
Right, inferior frontal gyrus, BA 47	21	3.03	33	20	−20	0.001

ADHD girls-TD girls						
*No voxel activated*						

*Note*. Minimum cluster threshold = 10 voxels; TD, typically developing youth; ADHD, youth with attention deficit and hyperactivity disorders; INCO-CO, contrast between incoherent and coherent conditions; MNI, Montreal Neurological Institute; BA, Brodmann Area; *K* (voxel) gives the number of voxels in each cluster; the *Z* value is the *Z*-score given for that specific cluster with its *p* value associated; *x*,  *y*,  *z* corresponds to the *x*, *y*, *z* MNI coordinates of the most central voxel of the cluster.
